# Transcriptome sequencing wide functional analysis of human mesenchymal stem cells in response to TLR4 ligand

**DOI:** 10.1038/srep30311

**Published:** 2016-07-22

**Authors:** Sun Hwa Kim, Amitabh Das, Jin Choul Chai, Bert Binas, Mi Ran Choi, Kyoung Sun Park, Young Seek Lee, Kyoung Hwa Jung, Young Gyu Chai

**Affiliations:** 1 Department of Molecular & Life Science, Hanyang University, Ansan, 15588, Republic of Korea; 2 Institute of Natural Science & Technology, Hanyang University, Ansan, 15588, Republic of Korea; 3 Department of Bionanotechnology, Hanyang University, Seoul, 04673, Republic of Korea

## Abstract

Due to their multipotentiality and immunomodulation, human mesenchymal stem cells (hMSCs) are widely studied for the treatment of degenerative and inflammatory diseases. Transplantation of hMSCs to damaged tissue is a promising approach for tissue regeneration. However, the physiological mechanisms and regulatory processes of MSC trafficking to injured tissue are largely unexplored. Here, we evaluated the gene expression profile and migratory potential of hMSCs upon stimulation with the TLR4 ligand lipopolysaccharide (LPS). Using RNA sequencing, we identified unique induction patterns of interferon stimulated genes, cytokines and chemokines involved in chemotaxis and homing. The −950 to +50 bp regions of many of these LPS-responsive genes were enriched with putative binding motifs for the transcription factors (TFs) interferon regulatory factor (IRF1) and nuclear factor kappa B (NF-κB1, REL), which were also induced by LPS along with other TFs. Chromatin immunoprecipitation assays showed that IRF1 bound within their target genes promoter region. In addition, IRF1 attenuation significantly down-regulated interferon stimulated genes as well as key cytokines. Furthermore, using pharmacological inhibitors, we showed that the NF-κB and phosphatidylinositol 3-kinase (PI3K) pathways regulate the migratory and cytokines/chemokines response to LPS. These unprecedented data suggest that IRF1 and NF-κB orchestrate the TLR4-primed immunomodulatory response of hMSCs and that this response also involves the PI3K pathway.

Mesenchymal stem cells (MSCs) are adherent, fibroblast-like, multipotent, nonhematopoietic cells harboring numerous features that make them candidates not only for cell-based regenerative and repair therapy but also for immunotherapy. They are multipotent adult stem cells that have the capacity for self-renewal and, under appropriate conditions, differentiate into mesenchymal-type cells (adipocytes, osteoblasts, and chondrocytes) as well as into myocytes, neurons, endothelial cells, astrocytes, and epithelial cells^1^. In addition to their differentiation potential, MSCs play a pivotal role in regulating the immune system in a manner that depends on their state of activation^2^. Given this information, MSCs secrete a variety of factors with proinflammatory, immunosuppressive, or antiviral and anti-inflammatory effects^3^. For example, previous studies reported that MSCs recruited and/or activated neutrophil granulocytes *via* the release of interleukin (IL)-6 and IL-8, interferon β (IFNβ), granulocyte-macrophage colony-stimulating factor (GM-CSF), or macrophage migration inhibitory factor (MIF)^4^,^5^. In addition, MSCs are able to produce indolamine 2,3-dioxygenase (IDO), transforming growth factor β (TGFβ), prostaglandin E2 (PGE2), cyclooxygenase 2 (COX2), or human leukocyte antigen G5 (HLA-G5) to inhibit effector T-cell immunity^6^,^7^,^8^. These results suggest that MSCs possess an immune plasticity regarding inflammation and immunomodulation. However, the mechanisms mediating and guiding the plasticity of MSCs remain poorly understood.

MSCs have been shown to express active Toll-like receptors (TLR), which may modulate stem cell function^9^,^10^. Among them, TLR3 and TLR4 were consistently highly expressed in MSCs^10^. TLR4 induction by myeloid differentiation factor 88 (MyD88)-dependent signaling pathways activates downstream effectors including NF-κB, mitogen-activated protein kinase (MAPK), and PI3K, which ultimately induces inflammatory cytokine production^11^,^12^. A remarkable attribute of MSCs is that they migrate to areas of injury and to tumors, which has encouraged their investigation as therapeutic tools^9^,^13^. For instance, systemically administered MSCs have been shown to enhance improvement in animal models of stroke and myocardial infarction^14^,^15^. In addition, MSCs are very attractive candidates for targeted delivery of therapeutic gene products to the tumor microenvironment in animal models^16^. Although the migratory behavior of MSCs has now been addressed, distinct signals that affect the migration of MSCs are unknown. Therefore, a better understanding of the precise molecular mechanisms governing MSC homing may permit efficient targeted delivery of MSCs to desired sites of engraftment. We hypothesized that gene expression profiling of TLR4-primed MSCs would provide clues to the molecular pathways involved in MSCs migration.

In the present study, we therefore performed gene array and comparative gene expression profiling of hMSCs that were treated with the well-characterized TLR4 ligand lipopolysaccharide (LPS)^17^. To this end, we used RNA sequencing (RNA-seq), a technique that, unlike microarrays, provides unbiased profiling and the ability to identify novel transcribed regions and can be extremely accurate if a sufficient level of coverage is obtained^18^,^19^. Validation techniques, such as quantitative real-time RT-PCR (qRT-PCR)^20^, have corroborated the accuracy of RNA-seq.

This study is to apply a comprehensive RNA-seq method to assess differential gene expression associated with hMSCs migration. Our results show that TLR4-primed hMSCs and unstimulated control groups express chemotaxis- and inflammatory response-related genes in a differential manner, allowing a better understanding of the modes of action of TLR4. Furthermore, we show that TLR4 stimulation particularly promotes hMSCs migration capabilities through the NF-κB and PI3K pathways. Overall, the results deliver valuable information on molecular mechanisms behind hMSCs and TLR4-primed chemokines for stem cell migration, which will help to understand and utilize their functional plasticity in inflammation and immunomodulation.

## Results

### Morphological characterization, identification, and differential potential of hMSCs

**Figure 1:**
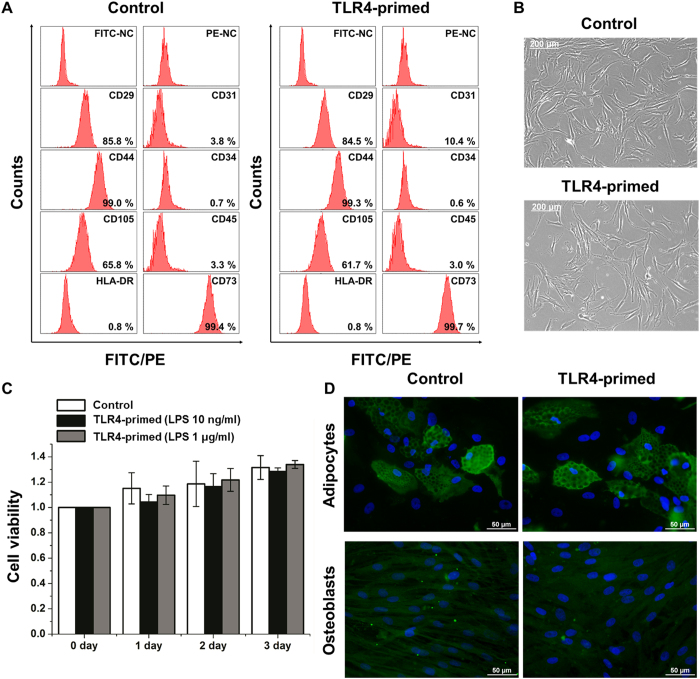
Immunophenotype, morphology and differential potential of TLR4-primed hMSCs. (**A**) Flow cytometric analysis demonstrates that hMSCs (passage 5) are positive for expression of the antigens CD29, CD44, CD73 and CD105. (**B**) No morphological changes were observed in TLR4-primed vs. control hMSCs. Original magnifications: X100. (**C**) Cell viability of hMSCs cultured for 1, 2, and 3 days after 4 hr LPS (10 ng/ml and 1 μg/ml) exposure using a WST assay. Vertical axis represents the relative absorbance at 450 nm. (**D**) hMSCs were cultured for 21 days under adipogenic and osteogenic differentiation conditions and then stained with FABP4 and osteocalcin antibodies (green) for adipocytes and osteoblasts, respectively. Nuclei were counterstained with DAPI (blue). Original magnifications: X400.

First, we characterized the immunophenotype and differentiation potential of non-hematopoietic BM stromal cells. To verify their identity, we analyzed the expression of typical MSC-related surface antigens by flow cytometry. As expected, the hMSCs were positive for the CD29, CD44, CD73 and CD105 after 5 passages (Fig. 1A). However, the hMSCs were negative for hematopoietic lineage markers CD31, CD34, CD45 and HLA-DR. When incubated for 4 hr with LPS, the hMSCs and their non-stimulated controls displayed a similar spindle shaped fibroblastic morphology (Fig. 1B). Thus, no morphological effects or cytotoxicity were visible. In addition, cell viability was investigated using the WST assays of LPS in the hMSCs. The viability of these cells did not change after 4 hr LPS (10 ng/ml and 1 μg/ml) exposure (Fig. 1C). Furthermore, after culturing under adipogenic and osteogenic differentiation conditions for 21 days, TLR4-primed and control hMSCs exhibited similar differentiation potentials (Fig. 1D). These data indicate that TLR4-primed hMSCs maintain the typical characteristics of hMSCs.

### Gene-induction patterns in TLR4-primed hMSCs

**Figure 2:**
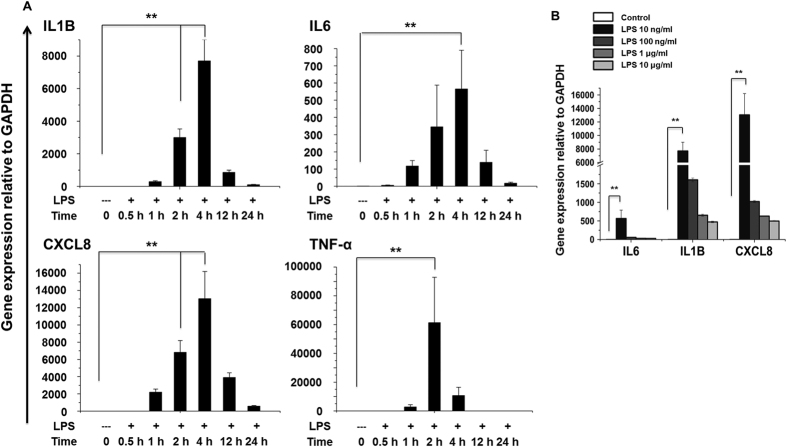
Induction of inflammatory response-related genes in TLR4-primed hMSCs. (**A**) Quantitative real-time reverse transcriptase PCR analysis of the expression of inflammatory genes in hMSCs stimulated with LPS (10 ng/ml). The expression of inflammatory genes was significantly up-regulated at the indicated times in cells treated with LPS compared to untreated cells (**P < 0.001). (**B**) hMSCs were stimulated with different doses of LPS (10 ng/ml to 10 μg/ml) for 4 hr before analysis of inflammatory response-related genes by quantitative real-time reverse transcriptase PCR analysis. Gene expression was normalized to GAPDH transcript levels. The data represent three independent experiments. The values are the mean ± SD of triplicate wells. **P < 0.001.

To determine the proper time course responses, we performed an expression analysis in TLR4-primed vs. control hMSCs. LPS (10 ng/ml) caused a transient up-regulation of key inflammatory response-related genes, peaking at 2 hours (hr) for TNFα and at 4 hr for IL1β, IL6, and CXCL8 (Fig. 2A). To assess the effects of low and high doses of LPS on the expression of key inflammatory response-related genes (IL1β, IL6 and CXCL8), hMSCs were treated with a low-dose LPS (10 ng/ml) or a high-dose LPS (100 ng/ml to 10 μg/ml) for 4 hr. As shown in Fig. 2B the magnitudes of expression induced by low-dose LPS (10 ng/ml) were significantly high compared with high-dose LPS. We hence used the lower doses for subsequent analyses. These lower doses of LPS (10 ng/ml) were also used in previous studies^4^,^21^,^22^ investigating the general induction patterns of MSCs through LPS.

### Differential gene expression profiling of hMSCs exposed to TLR4 ligand using RNA-seq analysis

To gain insight into the molecular consequences of LPS treatment, RNA-seq experiments were performed. Based on the results shown in Fig. 2, we selected a 4 hr time point for whole-genome transcriptional profiling. Three independent samples (biological replicates) for each treatment were processed. Six libraries obtained from control (3 samples) and TLR4-primed hMSCs (3 samples) treatments were sequenced. We used a 1% false discovery rate (FDR), *P* ≤ 0.001, and fold change ≥1.0 log_2_ for up- or down-regulation as the criteria for defining differentially expressed genes.

**Figure 3:**
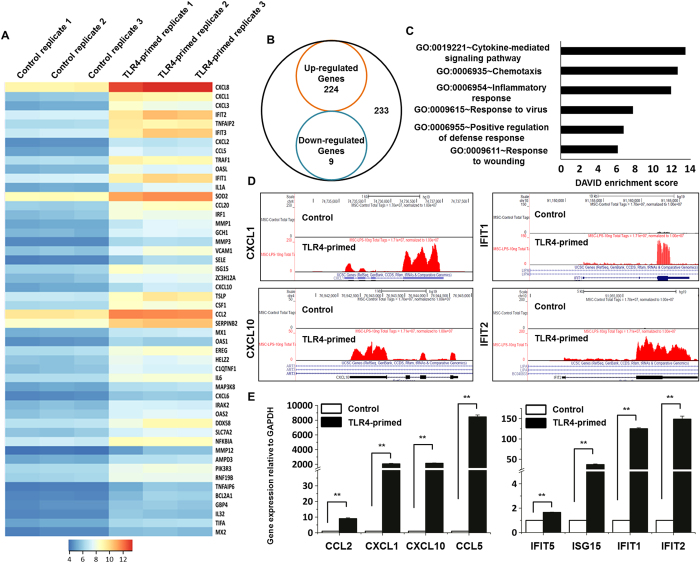
RNA-seq analysis reveals differentially expressed genes in TLR4-primed hMSCs. (**A**) Heat map of the top 50 up-regulated chemotaxis/inflammatory genes in TLR4-primed (4 hr) vs. control hMSCs. *P* < 0.001, fold change ≥1.0 log_2_ for the significant determination of differential expressed genes. Each row shows the relative expression level for a single gene, and each column shows the expression level of a single sample. Biological replicates (n = 3) for each condition were performed. (**B**) Venn diagram displaying the number of inducible or repressible (≥1.0 log_2_-fold change) genes after LPS treatment. (**C**) Gene Ontology analysis of functional annotations (biological process) up-regulated by LPS (4 hr) treatment. (**D**) UCSC Genome Browser images representing normalized RNA-seq read densities in TLR4-primed (4 hr) vs. control hMSCs. (**E**) Confirmation of differentially expressed genes (cytokines/chemokines and interferon stimulated genes) by quantitative reverse transcription-polymerase chain reaction in TLR4-primed hMSCs (**P < 0.001). The data represent three independent experiments. The values are the mean ± SD of triplicate wells.

By the above criteria, 233 genes were differentially regulated following LPS treatment at 4 hr, of them, 224 genes were up-regulated, whereas 9 genes were down-regulated (Fig. 3B). Interestingly, we found five chemokines among the top 10 differentially expressed genes, namely chemokine (C-X-C motif) ligand 8 (CXCL8), chemokine (C-X-C motif) ligand 1 (CXCL1), chemokine (C-X-C motif) ligand 3 (CXCL3), chemokine (C-X-C motif) ligand 2 (CXCL2), and chemokine (C-C motif) ligand 5 (CCL5) (Fig. 3A,D and Table 1). We next performed functional classification analyses of the up-regulated genes using DAVID Informatics Resources^23^ through classification into gene ontology (GO) categories (FDR 0.01) based on biological process (BP) and molecular function (MF) categories. The genes up-regulated in response to TLR4-primed hMSCs were involved in several BPs and MFs. Importantly; the largest groups of genes were involved in cytokine-mediated signaling pathways, chemotaxis, and inflammatory responses. Other pathways, such as the regulation of response to virus, positive regulation of defense response and response to wounding, were also identified (Fig. 3C). In addition, we confirmed the induction of key chemokines as well as antiviral immunity and signaling genes, including CCL2, CCL5, CXCL1, CXCL10, IFIT1, IFIT2, IFIT5 and ISG15 (Fig. 3E). As the down-regulated genes were not associated with inflammation, only the up-regulated genes were further studied. We confirmed by GO analysis (FDR 0.01) using DAVID Bioinformatics Resources that TLR4-primed down-regulated transcripts were associated with regulation of nucleosome and chromatin assembly in hMSCs (Supplementary Figure S1).

### Gene network, molecular and cellular functional analysis following TLR4-primed hMSCs

**Figure 4:**
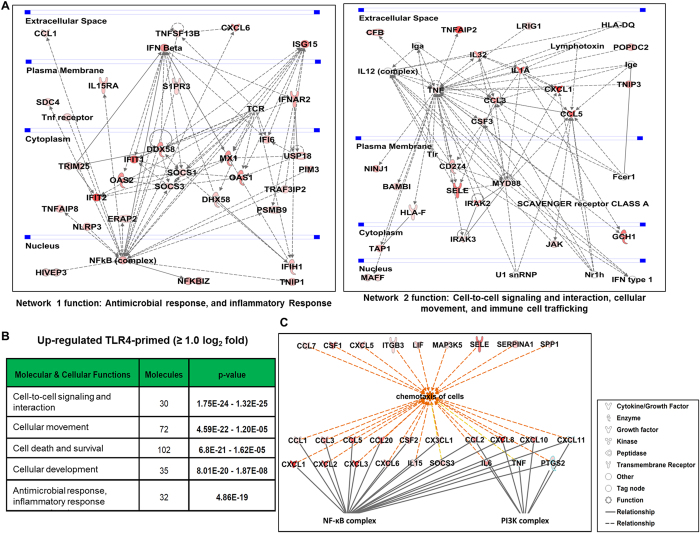
Top IPA-based network involved in chemotaxis and canonical pathway analyses in TLR4-primed hMSCs. (**A**) Ingenuity^®^ Bioinformatics pathway analysis of gene networks displaying interactions between genes related to antimicrobial response, inflammatory response, cell-to-cell signaling and interaction, cellular movement, and immune cell trafficking that were differentially expressed in TLR4-primed hMSCs. (**B**) The most highly represented molecular and cellular functions for differentially expressed genes in TLR4-primed hMSCs. (**C**) The chemotaxis molecules were highly connected, namely, NF-κB complex and PI3K complex, was assessed using the IPA molecule activity predictor.

Next, we performed IPA^24^ to identify gene networks that represent the intermolecular connections among interacting genes based on functional knowledge inputs. These pathways have the potential to define molecular targets associated with TLR4-priming in hMSCs. The IPA analysis revealed top networks of the differentially expressed genes established at 4 hr of LPS treatment. Network 1 and 2 is illustrated in Fig. 4A. Interestingly, the genes in network 1 and 2 were mainly involved in antimicrobial, inflammatory response, cell-to-cell signaling and interaction, cellular movement, and immune cell trafficking. Importantly, these genes formed a network with the NF-κB complex as the central node. To further functionally characterize the TLR4-primed hMSCs, the TLR4-primed genes were functionally annotated; in this way we found that cell-to-cell signaling and cellular movement were among the top five affected molecular and cellular functions (Fig. 4B). The most pronounced functional network over-represented in these data involved the NF-κB and PI3K complexes, which are the central molecules of an interconnected regulatory system. The data suggest that the NF-κB complex and the PI3K complex orchestrate a host of proinflammatory chemokines (CCL1, CCL2, CCL3, CCL5, CCL7, CX3CL1, CXCL2, CXCL3, CXCL6, CXCL8, CXCL10, IL6, PTGS2, TNF-α) to mediate signaling and chemotaxis in TLR4-primed hMSCs (Fig. 4C).

### Differential expression of TFs and signaling pathways modulated through TLR4-primed hMSCs

**Figure 5:**
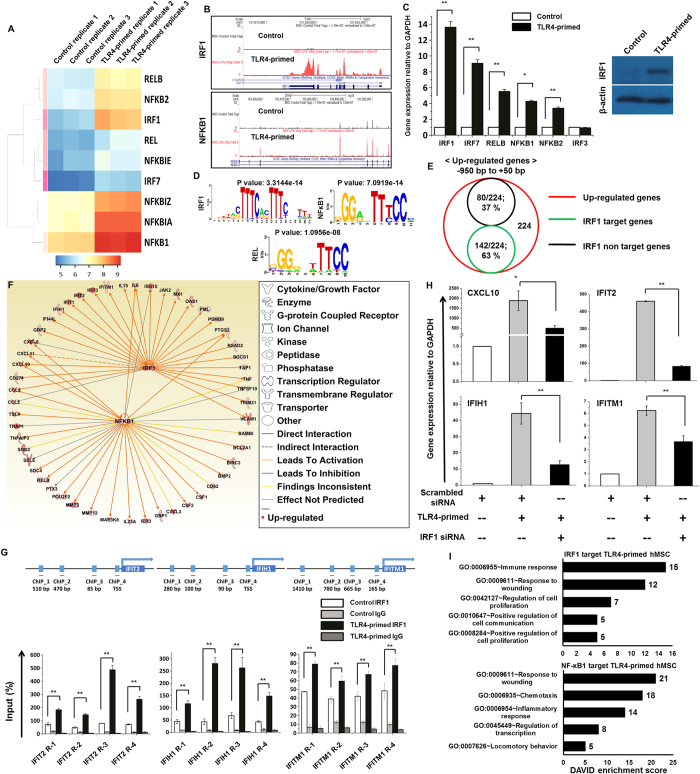
Transcriptomic analyses of selected TF families in TLR4-primed hMSCs. (**A**) Heat map representing differential expression of NF-κB TF families IRF1, and IRF7. (**B**) UCSC Genome browser images representing normalized RNA-seq read densities for TF expression in TLR4-primed vs. control hMSCs. (**C**) Confirmation of differentially expressed TFs by quantitative reverse transcription-polymerase chain reaction and western blotting in TLR4-primed hMSCs (*P < 0.01 and **P < 0.001). The data represent three independent experiments. The values are the mean ± SD of triplicate wells. (**D**) Patterns of transcription factor motif enrichments within the promoters of the LPS-stimulated genes. (**E**) Venn diagrams of up-regulated genes associated with IRF1 in TLR4-primed hMSCs. (**F**) The activity of highly connected positive regulators of the chemotaxis genes IRF1, and NF-κB1 led to the activation of this network, as assessed using the IPA molecule activity predictor in TLR4-primed hMSCs. (**G**) ChIP assay to determine the presence of IRF1 at selected genes. The ChIP-enriched samples were analyzed using quantitative PCR with primers targeting the sub-regions of the selected genes. The IRF1 occupancy is increased following LPS exposure at the sub-regions of the selected genes promoters. The graphs represent the mean values of enrichment relative to input DNA from three independent experiments. **P < 0.001 compared with the control. (**H**) Attenuation of IRF1 significantly decreased interferon stimulated genes and chemokines by quantitative reverse transcription-polymerase chain reaction. Gene expression was normalized to GAPDH transcript levels. *P < 0.01 and **P < 0.001 compared with the control. The data represent three independent experiments. (**I**) Results of the GO term analysis using DAVID on genes that were regulated by NF-κB1 and IRF1 in TLR4-primed hMSCs.

In addition to differentially expressed cytokines/chemokines, the annotation of the RNA-seq data also revealed that two families of TFs were significantly up-regulated at 4 hr TLR4-primed hMSCs. These TFs, including IRF and NF-κB, are important in inflammatory diseases. We observed that NF-κB1, NF-κB2, REL, RELB and IRF1 were significantly up-regulated in TLR4-primed hMSCs (Fig. 5A,B). However, other members of the IRF or STAT families were unaffected (Supplementary Figure S2), again suggesting that TLR4-priming affects TFs in a highly selective manner. In addition, we confirmed the induction of key TFs by quantitative reverse transcription-polymerase chain reaction and western blotting (Fig. 5C). Next, we conducted a TF motif analysis to assess TLR4-primed gene expression in hMSCs. We used the Pscan software tool^25^ to perform the *in silico* computational analysis of over-represented *cis*-regulatory elements within the 5′-promoter regions of coordinately regulated genes. Applying this score to the promoters of the genes differentially expressed (≥1.0log_2_-fold) at 4 hr in response to TLR4-priming revealed that the putative binding sites for IRF1, and NF-κB1, REL were significantly enriched (Fig. 5D). Furthermore we analyzed how many TLR4-primed up-regulated genes contain an IRF1 binding motif in the promoter sequence. Among up-regulated genes we found a significant percentage (142/224; 63%) had an IRF1 binding motif in the promoter region (from −950 bp to +50 bp) and are summarized in Fig. 5E and Supporting Information Table 1. In addition to TF motif analysis, we also applied IPA software^24^ to identify the target genes that were directly or indirectly activated by identified TFs in response to TLR4 stimulation. Importantly, we found that the expression of most cytokines and chemokines was directly regulated by the identified TFs, including IRF1 and NF-κB1 (Fig. 5F and Supporting Information Table 2). Further we hypothesized that IRF1 would also associate one or more sub-regions of the target genes promoter. Accordingly, we performed chromatin immunoprecipitation (ChIP) studies to map IRF1 occupancy along the target genes promoter region in TLR4-primed hMSCs. As shown in Fig. 5G IRF1 bind to four distinct IFIH1, IFIT2 and IFITM1 promoter sites, and their relative abundance paralleled one another. Next, we investigated whether IRF1 depletion (Supplementary Figure S3) affects IRF1 target genes. Using real-time RT-PCR analysis we found that compared to scrambled siRNA control (NC), IRF1 target genes were increased following TLR4-primed and IRF1 depletion cells led to significant reduction in IRF1 target genes, as well as CXCL10 cytokines (Fig. 5H). To further functionally classify the IRF1- and NF-κB1-regulated genes, they were functionally annotated using DAVID 6.7 software package^23^. Interestingly, we observed strong enrichments of GO terms for the NF-κB1 and IRF1-regulated transcripts associated with the response to wounding and immune response in TLR4-primed hMSCs (Fig. 5I). Taken together, these findings suggest that NF-κB1 and IRF1 might be involved in the regulation of chemotaxis response in TLR4-primed hMSCs.

### NF-κB and PI3K pathways regulated TLR4-primed hMSCs migration

**Figure 6:**
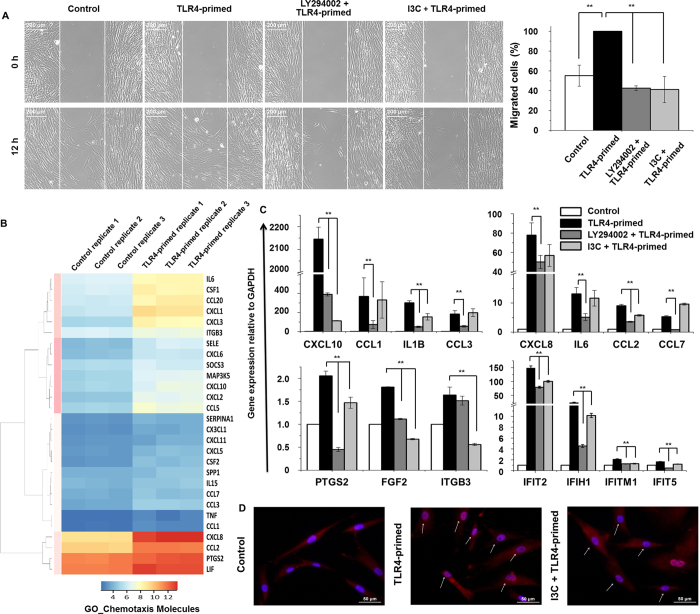
Effects of PI3K and NF-κB signaling inhibitors on TLR4-primed cell migration and gene expression. (**A**) The migration of hMSCs was measured using a wound scratch assay. Representative images of migrating hMSCs after 12 hr in the presence or absence of LPS (10 ng/ml), LY294002 (20 μM), and I3C (1 mM). Also shown is the number of hMSCs that migrated into the middle blank fields. There was a significant difference between the TLR4-primed and PI3K, NF-κB signaling pathway inhibitor-treated groups in terms of the speed of wound healing when compared with the control group (**P < 0.001). (**B**) Heat map representing chemotaxis/inflammatory genes expression at 4 hr in TLR4-primed hMSCs compared with controls. *P* < 0.001, fold change ≥1.0 log_2_ for the significant determination of differential expressed genes. (**C**) Differential effects of I3C (1 mM) and LY294002 (20 μM) on the expression of TLR4-primed cytokines/chemokines and interferon stimulated genes. CXCL10, IL1B, CCL2, PTGS2, FGF2, IFIT2, IFIT5, IFIH1 and IFITM1 were down-regulated by both inhibitors. CCL1, CCL3, CXCL8, IL6, and CCL7 were down-regulated by LY294002 only, and ITGB3 was down-regulated by I3C only. Gene expression was normalized to GAPDH transcript levels. **P < 0.001 compared with the control. The data represent three independent experiments. (**D**) Effects of LPS and I3C on the subcellular localization of NF-κB as determined by immuno-fluorescence microscopy. Original magnifications: X400.

Stem cell migration to the wound site is a prerequisite for wound restoration^26^. To identify the possible role of TLR4 in the process of hMSCs homing, control cells and TLR4-primed hMSCs were compared. LPS treatment significantly increased the number of hMSCs that migrated into a “wound field” within 12 hr; however, the increase was not observed after addition of the NF-κB and PI3K pathways inhibitors, Indole-3-Carbitol (I3C, 1 mM) and LY294002 (20 μM), respectively (Fig. 6A). To further functionally classify the chemotaxis-regulated genes, they were functionally annotated using DAVID 6.7 software package. In this way, we found that the majority of the TLR4-primed cytokines/chemokines transcripts were associated with the chemotaxis response (Fig. 6B). To probe again whether NF-κB and PI3K triggered deregulation of chemotaxis-related genes, we measured the expression of selected chemotaxis-related genes upon exposure to I3C and LY294002. We found that I3C and LY294002 significantly down-regulated the expression of key chemotaxis-related and interferon stimulated genes in TLR4-primed hMSCs (Fig. 6C). Furthermore, immunofluorescence studies revealed an increased nuclear translocation of NF-κB upon TLR4-primed, which was prevented by I3C treatment (Fig. 6D). Taken together, these data strongly indicate that NF-κB and PI3K kinase might be involved in the regulation of chemotaxis response in TLR4-primed hMSCs.

## Discussion

Use of the multipotentiality and immunomodulatory properties of MSCs is a credible strategy for the treatment of tissue injury and immune-related disorders^3^,^27^. MSCs could play roles in inflammation-associated conditions (such as infections or tissue injury) and confirm the immunomodulatory plasticity of MSCs anticipated by previous studies^3^. Recently, other laboratories reported that TLR4 is highly expressed in MSCs, which may modulate stem cell function and be used for a variety of therapeutic applications^9^,^10^. For instance, MSCs reduce lung injury caused by systemic^28^ or intratracheal LPS challenge^29^. One study also reported that hMSCs attenuated TLR4-primed injury in explanted human lungs^30^. However, none of these studies addressed the effects of the TLR ligand LPS; therefore, the present study focused on those responses using RNA-seq analysis in hMSCs. Notably, these data were accurate, although only three independent biological replicates for each sample were used. In addition, we investigated the roles of IRF1 TFs and IRF1 TF-regulated genes in TLR4-primed hMSCs. Finally, we showed that TLR4-priming promotes hMSCs migration capabilities through NF-κB and PI3K pathways.

The RNA-seq analysis revealed that the genes associated with chemotaxis and inflammatory response were significantly up-regulated in TLR4-primed hMSCs. Both the extent of the fold-change and the number of genes were significantly modulated. The following chemotaxis- and inflammatory response-related genes were markedly affected after TLR4-priming: cytokines or chemokines (CCL1, CCL2, CCL3, CCL4, CCL7, CX3CL1, CXCL2, CXCL3, CXCL5, CXCL6, CXCL8, CXCL10); interleukin and interleukin-related genes (IL1A, IL1B, IL6, IL15, IL15RA, IL23A, IL32, IL34, IRAK2, LIF); TNF and TNF-related genes (TNF, TNFAIP2, TNFAIP3, TNFAIP6, TNFAIP8, TNFRSF9, TNFSF9); a prostaglandin-related gene (PTGS2); and interferon-related genes (IFI6, IFI44, IFI44L, IFIH1, IFIT1, IFIT2, IFIT3, IFIT5, IFITM1, IRG1, ISG15, ISG20, MX1, MX2, OAS1, OAS2, OAS3, OASL) (Fig. 3A).

We found that TLR4-priming significantly induced the expression of key cytokines or chemokines in hMSCs. Chemotactic cytokines are key regulators of migratory processes in hematopoietic and non-hematopoietic cells^31^. These molecules regulate cell trafficking and localization in tissue compartments and play a significant role in cell activation, differentiation and survival. For example, they affect leukocyte rolling^32^. A previous study reported that CXCL2, CXCL3, CXCL6, and CXCL8 play a crucial role in the migration of leukocytes from the bone marrow, as well as rolling and tight adhesion formation and transmigration^33^. In particular, Ringe *et al*. reported that CXCL8 induced the migration of human MSCs *in vitro*^34^. In addition, CXCL8 can induce migration of hMSCs to ischemic brain *in vitro*^35^. CXCL6 is tightly regulated in MSCs. It was demonstrated that CXCL6 is stimulated by LPS^36^, and CXCL2 provokes a time- and dose-dependent increase in leukocyte rolling and adhesion^37^. CXCL10 plays a role in inflammatory demyelinating diseases by facilitating leukocyte trafficking in the brain and contributing to the destruction of the myelin sheath or neurons^38^. Furthermore, IL6 and LIF can be effective activators of immune cells and may chemotactically recruit cells to sites of inflammation. For instance, IL6 induced the migration of T cells^39^, and LIF has been shown to recruit peritoneal macrophages^40^. These patterns are consistent with our studies showing the effects of TLR4-priming on the induction of cytokines/chemokines in hMSCs (Fig. 3A–E).

TLR4 could activate various signaling pathways, including phosphoinositide 3-kinase, Jun N-terminal kinase (JNK), p38, NF-κB, extracellular signal-related kinase, and IRF3 leading to the induction of numerous target genes involved in antiviral immunity, including IFN-β and CXCL10^41^. In the present study, we established the profound up-regulation of several genes involved in antiviral immunity and signaling, such as IFI44, IFI44L, IFIH1, IFIT2, IFIT5, IFITM1, IRG1, ISG15, ISG20, MX1, MX2, OAS2 and OAS3 in TLR4-primed hMSCs (Fig. 3A,E). This result suggests that TLR4-priming caused the activation of IFN signaling-pathway-induced gene expression in hMSCs, although the modulation of IFN-α/β genes was not detected in the RNA-seq analysis. A previous study reported that TLR3-primed infection up-regulated the expression of IRF3 in mouse MSCs^42^. Unexpectedly, we were unable to identify IRF3 and its target gene IFN-β (Supporting Information Figure S2), but not CXCL10, in TLR4-primed hMSCs. However, because no IRF3 activation could be observed in hMSCs, the mechanism by which the production of CXCL10 and IRGs is regulated in these cells remains unclear. This mechanism is the subject of ongoing investigations. Importantly, by using RNA-seq we first identified IRF1 to be significantly up-regulated in TLR4-priming (Fig. 5A–C). A previous study reported that IRF1 could be a master transcription factor contributing to IRGs^43^. However, IRF2, IRF4, IRF6, IRF8 were unaffected by TLR4-priming, suggesting highly selective induction of TFs through TLR4-priming in hMSCs. In addition to IRF1 we identified NF-κB TFs (RELB, REL, NF-κB1) (Fig. 5A–C). Similar observations were also made in mouse MSCs stimulated with TLR3 ligand, revealing the strong up regulation of NF-κB^42^.

To further delineate conserved transcription factor-binding motifs, we performed TF motif analysis on TLR4-primed hMSCs. The core promoters of co-expressed genes (typically, regulatory regions within −1000 to +50 bp relative to the transcriptional start site) can be evaluated for overrepresented *cis*-regulatory elements after partitioning into suitable modules. Among the two ranges available in Pscan that are closest to this region of interest (−950 to 50 and −1000 to 0), the −950 to +50 bp range was selected for the analyses. We found that the promoters of differentially expressed genes were enriched for putative NF-κB1, REL and IRF1 binding sites (Fig. 5D). These analyses provide the first insights into TF binding motifs that was involved in regulating subset specific genes in TLR4-primed hMSCs (Fig. 5E). Next, we used IPA software to identify the target genes that were directly or indirectly activated by the identified TFs (i.e., NF-κB1 and IRF1) in TLR4-priming. Importantly, we found that the expression of the majority of the TLR4-priming-induced cytokines/chemokines was directly regulated by the identified TFs (Fig. 5F and Supporting Information Table 2). In addition our results show that IRF1 depletion affected IRF1 target genes, including CXCL10 cytokines (Fig. 5H). Taken together, our results suggest that IRF1 has a unique role in TLR4-mediated IFN signaling-as well as cytokines/chemokines expression.

One of the most striking features is that our RNA-seq analysis identified several novel patterns of differential gene expression in TLR4-primed vs. untreated hMSCs. In particular, this technology allowed us to identify BMP2, FGF2, ITGB3, MMP1, MMP3, MMP12 and MMP13 TLR4-primed genes in hMSCs. Most interestingly, other members of the BMP, ITGB, or MMP like BMP6, ITGB1, and MMP2 families were unaffected by TLR4-priming (Supplementary Figure S2), suggesting the highly selective induction of those genes in hMSCs. ITG is highly expressed in bone marrow derived MSCs including ITGA4 and ITGB1^44^, and previous studies revealed that ITGB3 plays a critical role in endothelial cell migration^45^. FGF can mediate migration activity of MSCs^46^. BMP2 was originally recognized as an osteoinductive cytokine and has a potential role in cell migration, proliferation, and differentiation^47^. Additionally, recently Chu *et al*. reported that BMP2 deletion attenuated proliferation and migration rates of lung cancer samples^48^. Nevertheless, further studies are warranted how these TLR4 target genes are expressed as well as their role on migratory activity in hMSCs.

Among all TLRs, the common signaling feature is the activation of the NF-κB TFs, which have been implicated in controlling the expression of inflammatory cytokines and cell maturation molecules^10^. A previous study reported that cultured MSCs express TLR molecules 1 to 8, and activation of MSCs by TLR ligands induced IL6 secretion and NF-κB nuclear translocation^12^. In our study both NF-κB and PI3K pathways were also crucial for TLR4-primed hMSCs. Dumitru *et al*. demonstrated that p38-MAPK is an important regulator of TLR3-induced cytokine release by mouse MSCs^42^. Surprisingly, in the present study, we found that not only NF-κB but also PI3K is a potential regulator of TLR4-induced cytokine release and hMSCs migration (Fig. 6A,D). Furthermore, the most pronounced functional network over-represented in these data involved both the NF-κB and PI3K complexes, which form the central molecules of an interconnected regulatory system, suggesting that these two complexes connected proinflammatory cytokines that mediate the signaling and induction of chemotaxis of TLR4-primed hMSCs (Figs 4C and 6C).

One of the most intriguing features of MSCs is that they are immunosuppressive and inhibit function of immune cells including T cells, B cells, macrophages/monocytes, natural killer cells, neutrophils, and dendritic cells^2^,^49^,^50^,^51^,^52^. Consequently, MSCs are currently being widely investigated for their potential use in the treatment of numerous inflammatory diseases demonstrating an efficient protection against allograft rejection, graft-versus-host disease, experimental autoimmune encephalomyelitis, collagen-induced arthritis, sepsis, and auto immune myocarditis^7^,^30^,^49^,^53^. Importantly, it is believed that during MSC-based cellular therapy, huge numbers of bone marrow-derived MSCs (BM-MSCs) are delivered systemically to the peripheral blood for the effective treatment of those diseases. Interestingly, the detection of TLRs expression by MSCs recently encouraged scientific and clinical communities to explore the possible link regarding the potential effects of TLR signaling on MSCs biology as they have been linked to the perpetuation of inflammatory diseases that MSCs will likely encounter in the sites of injury^10^. In this situation, upon LPS (a representative TLR4 agonist) challenge MSCs could promote an anti-inflammatory response from the bloodstream to the tissue site of infection. Recently, other laboratories reported that TLR4 is highly expressed in MSCs, which may modulate stem cell function and be used for a variety of therapeutic applications^9^,^10^. Furthermore several studies have demonstrated beneficial effects of MSCs treatment in animal models of LPS-induced sepsis or lung injury^7^,^53^. This hypothesis is further supported by previous results showing that TLR4 engagement enhances MSCs-mediated immunosuppressive properties of human BM-MSCs^54^. Nevertheless further studies are warranted to establish the potential role of TLR signaling in migration and bio distribution of MSCs *in vivo*, which is of great clinical relevance. Therefore, TLR4 stimulation induced by LPS may be one mechanism that specifically drives the immune modulating functions of the MSCs.

Overall, our RNA-seq data provide novel insights into the transcriptional landscape of TLR4-primed hMSCs, reflecting the robust and reliable kinetic development and modulation of cell reactivity during the early course of the inflammatory response. Regardless of certain boundaries in exactitude, TLR4-primed gene expression profiling in hMSCs, clustering, and the prediction of TFs offer valuable information for future studies, such as of potential gene targets for chromatin immunoprecipitation (ChIP)-seq assays. This model can subsequently be extended to include data from other high-dimensional surveys, such as microRNA, ChIP-seq, and proteomics, providing more advanced insight into global gene regulation and their role in hMSCs immunomodulation.

## Conclusion

This study is to comprehensively describe the gene expression profiles of TLR4-primed hMSCs. We identified unknown immune genes as well as cytokines and chemokines involved in the chemotaxis of TLR4-primed hMSCs. We further find that the NF-κB and PI3K pathways orchestrate the expression of these cytokines and chemokines as well as the migration of TLR4-primed hMSCs. Furthermore, we examined previously unidentified TFs (IRF1), NF-κB TFs (RELB, REL, NF-κB1) and TF-regulated genes especially IRF1 in TLR4-primed hMSCs. Thus, our findings elucidate novel mechanisms by which TLR4 modulate hMSCs behavior and, ultimately, provide a better understanding of the role of hMSCs in inflammation and immunomodulation.

## Methods

### Cell Culture, stimulation and morphological analysis of hMSCs

Experiments were performed using human bone marrow MSCs (hMSCs) which were derived from donor, a black 21 year old female, these cells were purchased from Lonza (donor 7F3915 Walkersville, MD) and cultured in low-glucose Dulbecco’s modified Eagle’s medium (DMEM) (Gibco, Carlsbad, CA) supplemented with 10% fetal bovine serum (FBS) (Hyclone, Logan, UT) and penicillin (100 U/ml)/streptomycin (100 mg/ml) (Gibco, Carlsbad, CA). The cells were maintained in a humidified incubator with 95% air and a 5% CO_2_ atmosphere at 37 °C. The medium was replaced every 3–4 days. In this study, MSCs were used at passages 5 or 6. The cells were incubated with LPS (10 ng/ml, Sigma-Aldrich, St. Louis, MO) for the specified time intervals under normal culture conditions. The NF-κB and PI3K signaling pathway inhibitors Indole-3-Carbitol (I3C) and LY294002, respectively, were purchased from Sigma-Aldrich (St. Louis, MO). Unless otherwise indicated, I3C and LY294002 were freshly dissolved before use in the experiments. In this study, we used 1 mM of I3C and 20 μM of LY294002^55^,^56^. hMSCs were cultured in poly-_D_-lysine-coated culture plates. On the following day, cells were treated for 4 hr with either conditioned medium (control) or LPS (“TLR4-primed” MSCs). The morphology of hMSCs (TLR4-primed) was analyzed for each independent experiment.

### Cell viability assay

Cell viability was assayed using a tetrazolium salt colorimetric assay using PreMixWST1 according to the manufacturer’s instructions (Takara BioInc., Shiga, Japan). Cells were seeded in 96-well plate’s in 100 ml and incubated for the indicated time. PreMixWST-1 was added as indicated and incubated for an additional 4 hr, and absorbance was measured at 450 nm.

### Differentiation of hMSCs into adipocytes and osteoblasts

An hMSCs functional identification kit (R&D systems, Minneapolis, MN) was employed to verify hMSCs differentiation into adipocytes and osteoblasts. Briefly, hMSCs were cultured for 21 days in minimum essential medium (MEM, Gibco, Carlsbad, CA) containing adipogenic and osteogenic supplements. The medium was replaced every 3–4 days. After 21 days, differentiated cells were washed with phosphate-buffered saline (PBS) and fixed in cold 4% paraformaldehyde and incubated with primary antibodies directed against fatty acid binding protein 4 (10 μg/ml, R&D systems, Minneapolis, MN) for adipocytes or osteocalcin (10 μg/ml, R&D systems, Minneapolis, MN) for osteoblasts. The cells were washed and incubated with fluorescein-labeled anti-rabbit IgG (Jackson ImmunoResearch, West Grove, PA). Stained cells were observed using a microscope (Nikon, Tokyo, Japan).

### Flow cytometry analysis of hMSCs

Flow cytometry analysis was performed as described previously^57^. To stain the hMSCs, the cells were harvested by trypsin treatment and then washed with PBS and 2% normal serum for 5 min on ice. The cells were incubated for 30 min with fluorescein isothiocyanate (FITC)-conjugated antibodies against CD105, HLA-DR, CD29 (Serotec Ltd., Oxford, U.K), CD44 (Dakocytomation, Glostrup, Denmark), or with phycoerythrin (PE)-conjugated antibodies against CD34 (Serotec Ltd., Oxford, U.K), CD31, CD45 (DakoCytomation, Glostrup, Denmark), CD73, CD90 (BD Pharmingen, San Diego, CA). Control cells were prepared with FITC- and PE-mouse isotype control antibodies (Serotec Ltd., Oxford, U.K). The stained cells were analyzed with a FACS Calibur A (BD Bioscience, San Diego, CA).

### Total RNA isolation and cDNA library preparation for transcriptome sequencing (RNA-seq)

Total RNA was extracted using RNAiso Plus (Takara BioInc., Shiga, Japan) and QIAGEN RNeasy^®^ Mini kit (QIAGEN, Hilden, Germany)^58^. hMSCs or TLR4-primed hMSCs were completely lysed with RNAiso Plus, then 200 μl of chloroform was added, and the tubes inverted for 5 min. The mixture was centrifuged at 12,000 × *g* for 15 min at 4 °C, and the upper phase was placed into a new tube and 600 μl of 70% ethanol was added. The mixture was applied to an RNeasy mini column. The column was washed with wash buffer. To elute, RNase-free water (30 μl) was added directly onto the RNase mini column, followed by centrifugation at 12,000 × *g* for 3 min at 4 °C. For ribosomal RNA (rRNA) depletion in total RNA, the RiboMinus Eukaryote kit (Life Technologies, Carlsbad, CA) was employed according to the manufacturer’s instructions. RNA libraries were created using the NEBNext^®^ Ultra™ directional RNA library preparation kit for Illumina^®^ (New England BioLabs, Ipswich, MA). rRNA-depleted total RNA was fragmented into small pieces using divalent cations at elevated temperatures. The first-strand cDNA was synthesized using reverse transcriptase and random primers, and then second-strand cDNA synthesis was performed using DNA polymerase I and RNase H. The cDNA fragments were processed using an end-repair reaction after the addition of a single ‘A’ base, followed by adapter ligation. These products were purified and amplified through PCR to generate the final cDNA library. The cDNA fragments were sequenced using the Illumina HiSeq2000. Biological triplicate RNA sequencing was performed on independent RNA samples from the TLR4-primed hMSCs: control hMSCs 4 hr (3 samples) and TLR4-primed hMSCs 4 hr (3 samples).

### Differentially expressed gene analysis using RNA-seq data

FASTQ files from RNA-seq experiments were clipped, trimmed of adapters, and the low-quality reads were removed by the Trimmomatic^59^. Quality controlled FASTQ files were alignment to UCSC hg19 reference genome using the STAR (version 2.5.1) aligner software^60^ with three mismatches. To measure differential gene expression, DESeq2^61^ with the default parameters was used. A subset of condition-specific expression was defined as showing a 1.0log_2_-fold difference and *P*-adj < 0.001 in expression between controls and LPS treated samples. RNA-seq experiments were normalized and visualized using HOMER^62^ after preparing custom tracks for the UCSC Genome Browser (http://genome.ucsc.edu/). The acquired data were deposited in the Gene Expression Omnibus database under dataset accession no. GSE81478.

### Functional annotation

To functionally annotate the most significant genes, gene ontology analysis was performed by DAVID (Database for Annotation, Visualization and Integrated Discovery), version 6.7^23^. Gene ontology was analyzed using a modified Fisher’s exact *P*-value in the DAVID program. *P*-values less than 0.001 were considered greatly enriched in the annotation category.

### Graphical representation of networks and pathways

For generation of networks and pathways, the molecules from the filtered RNA-seq dataset were each mapped to following objects in Ingenuity’s Knowledge Base IPA (Ingenuity W Systems, Mountain View, CA)^24^. A fold-change cutoff of up- and down-regulated genes (≥1.0log_2_-fold change, *p*-value ≤ 0.05) was set to identify significantly and differentially regulated genes in TLR4-primed hMSCs. The graphical representation of molecular relationships between genes and gene products was based on genes or gene products, represented as nodes, and the biological relationship between two nodes was represented as an edge (line). All edges were supported by at least one reference from the literature, textbook or canonical information in Ingenuity’s Knowledge Base. The intensity of node color represented the degree of up-regulation (red). The nodes were displayed using shapes to represent functional classes of gene products.

### Transcription factor binding motif enrichment analysis

NCBI reference sequence mRNA accession numbers were subjected to transcription factor binding motif analysis using the web-based software Pscan^25^. The JASPAR^63^ database of TF binding sequences was analyzed using enriched groups of −950 base pair (bp) sequences to +50 bp of the 5′ upstream promoters. The range of −950 to +50 was selected from the range options in Pscan to obtain the best coverage for a −1000 to +50 bp range.

### Quantitative real-time RT-PCR

Reverse transcription of the RNA samples was performed as previously described^64^. Briefly, RNA was extracted from human MSCs using RNAiso Plus (Takara BioInc., Shiga, Japan) according to the manufacturer’s instructions. The total RNA was reverse-transcribed with PrimeScript reverse transcriptase (Takara BioInc., Shiga, Japan). The synthesized cDNA was amplified using SYBR Premix Ex Taq^TM^ II (Takara BioInc., Shiga, Japan). The sequences of primers are listed in Supporting Information Table 3. Primers were synthesized by GenoTech (Daejeon, Korea). Quantitative PCR cycling parameters were 95 °C for 10 min, followed by 40 cycles of 15 sec at 95 °C and 1 min at 60 °C with an ABI 7500 real-time PCR system (Applied Biosystems Inc., Carlsbad, CA). For comparative analysis, glyceraldehyde-3-phosphate dehydrogenase (GAPDH) was used as an internal control. The results were analyzed using the critical threshold (ΔCT) and the comparative critical threshold (ΔΔCT) methods in the ABI 7500 software with the NormFinder and the geNorm PLUS algorithms. The primers were obtained by Primer Bank (http://pga.mgh.harvard.edu/primerbank/index.html).

### Chromatin immunoprecipitation (ChIP) assay

ChIP experiments were performed using a protocol adapted from Upstate. Chromatin from 1 × 10^7^ cells was used for each immunoprecipitation. hMSCs were collected and resuspended in digestion buffer (50 mM Tris-Cl, pH 7.6; 1 mM CaCl_2_, 0.2% Triton X-100, 5 mM sodium butyrate, 1X protease inhibitor cocktail, 0.5 mM PMSF). Sonicate the lysate to shear DNA fragments ranging in size from 100–500 bp and keep the samples on ice. We used Bioruptor sonicator for four cycles of 10 minutes, with 30 seconds on/off. After sonication, the cells were incubated with RIPA buffer (10 mM Tris, pH 7.4, 1 mM EDTA, 0.1% SDS, 0.1% Sodium deoxycholate, 1% Triton X-100) and subjected to immunoprecipitation with antibodies against IRF1 (Cell Signaling, Danvers, MA; #8478) and normal rabbit IgG (Santa Cruz Biotechnology, Inc., Dallas, TX; sc#2025) used as a control with Dynabeads Protein A beads (Invitrogen, Waltham, MA) for 16 hr at 4 °C. The DNA was extracted and purified following Upstate’s instruction and used in regular PCR analysis. The chromatin immunoprecipitates for the proteins and IRF1 marks were analyzed using regular PCR, with one modification; the cDNA was replaced with immunoprecipitated DNA and normalized by input DNA. Primers used for ChIP-PCR are listed in Supporting Information Table 4.

### Immunocytochemistry

hMSCs were seeded onto coverslips in 4-well plates and cultured for one day. After LPS or inhibitor treatment, the cells were washed with PBS, fixed with 4% paraformaldehyde for 15 min and then permeabilized with cold methanol for 5 min. After blocking with 5% BSA in PBS for 1 hr, the cells were incubated with primary antibody overnight at 4 °C with anti-rabbit NF-κB p65 polyclonal antibody (1:200, Abcam, Cambridge, U.K) followed by incubation with an appropriate secondary donkey anti-rabbit IgG antibody (Jackson Laboratory, West Grove, PA) for 1 hr at room temperature. Following incubation, washing with PBS was performed three times and the cells were mounted with 4′, 6-diamidino-2-phenylindole (DAPI) containing mounting solutions (Vectashield, Vector Laboratories, Burlingame, CA) and imaged by immunofluorescence microscope (Nikon, Tokyo, Japan).

### Wound healing migration assay

The wound healing assay was performed according to the manufacturer’s instructions (Ibidi, Martinsried, Germany). Briefly, the cells were seeded into each well and incubated for 1 day. After LPS or NF-κB and PI3K signaling pathway inhibitor treatment, the insert was removed to create a “wound field”, and the cells were washed and incubated with 10% FBS-containing growth media for 12 hr. The cells were then visualized using a microscope (Leica, Wetzlar Germany).

### Knockdown of IRF1 gene expression by siRNA treatment

Oligonucleotide specific for IRF1 (ID # s7502) was from Ambion Applied Biosystems (Waltham, MA). IRF1 was attenuated with small interfering (si)RNA sense strand 5′-GCAGAUUAAUUCCAACCAAtt-3′ and antisense strand 5′-UUGGUUGGAAUUAAUCUGCat-3′ siRNA. MSCs cells were transfected using siPORT^TM^ NeoFX^TM^ transfection agent (Ambion Applied Biosystems, Waltham, MA; L/N: 1203023) with IRF1 siRNA constructs following the Silencer Select siRNA transfection protocol with non-targeting siRNAs (Ambion Applied Biosystems, Waltham, MA). IRF1 siRNA was used at concentrations of 25 nM for 48 hr using DMEM medium.

### Western blotting

The extraction of protein from the cells was performed using RIPA buffer [1% Triton X-100 in 50 mM phosphate buffer (pH 7.4)] containing a complete EDTA-free protease inhibitor cocktail (Roche Diagnostics, Basel, Switzerland). The extracted protein was separated on SDS polyacrylamide gels and transferred to polyvinylidene difluoride membranes (Schleicher & Schuell Bioscience, Inc., Keene, NH). The Western blot analysis was performed using anti-IRF1 (Cell Signaling, Danvers, MA; #8478) and anti-β-actin (Sigma-Aldrich, St. Louis, MO; A-5316) antibodies. β-actin was used as an internal control.

### Statistical analysis

Data are reported as means ± standard deviation of the mean (SD). Statistical analyses were performed using the SPSS 17.0 program (SPSS Inc., Chicago, IL). Data were analyzed using a one-way ANOVA, followed by Tukey’s honestly significant difference (HSD) post-hoc test; *P*-values < 0.05 were considered significant.

## Additional Information

**How to cite this article**: Kim, S. H. *et al*. Transcriptome sequencing wide functional analysis of human mesenchymal stem cells in response to TLR4 ligand. *Sci. Rep.* **6**, 30311; doi: 10.1038/srep30311 (2016).

## Supplementary Material

Supplementary Information

## Figures and Tables

**Table 1:** Top 50 up-regulated genes in TLR4-primed hMSCs.

Gene ID	Gene Name	log_2_ Fold Change
NM_000584	CXCL8	5.628353
NM_001511	CXCL1	5.171114
NM_002090	CXCL3	4.609657
NM_001547	IFIT2	4.402326
NM_006291	TNFAIP2	4.317706
NM_001031683	IFIT3	4.057472
NM_002089	CXCL2	3.822581
NM_001278736	CCL5	3.797388
NM_001190945	TRAF1	3.784328
NM_003733	OASL	3.736945
NM_001270930	IFIT1	3.624214
NM_000575	IL1A	3.601548
NM_000636	SOD2	3.576035
NM_001130046	CCL20	3.564536
NM_002198	IRF1	3.541069
NM_002421	MMP1	3.446365
NM_000161	GCH1	3.440226
NM_002422	MMP3	3.438683
NM_080682	VCAM1	3.334161
NM_000450	SELE	3.322095
NM_005101	ISG15	3.198972
NM_025079	ZC3H12A	3.179265
NM_001565	CXCL10	3.054366
NM_033035	TSLP	2.949228
NM_172212	CSF1	2.940032
NM_002982	CCL2	2.810485
NM_001143818	SERPINB2	2.806888
NM_001144925	MX1	2.784858
NM_016816	OAS1	2.693988
NM_001432	EREG	2.6927
NM_001037335	HELZ2	2.689742
NM_030968	C1QTNF1	2.681108
NM_000600	IL6	2.672508
NM_005204	MAP3K8	2.66725
NM_002993	CXCL6	2.63836
NM_001570	IRAK2	2.636229
NM_002535	OAS2	2.625638
NM_014314	DDX58	2.590368
NM_003046	SLC7A2	2.571215
NM_020529	NFKBIA	2.563756
NM_002426	MMP12	2.533638
NM_001025390	AMPD3	2.488023
NM_003629	PIK3R3	2.485976
NM_001127361	RNF19B	2.471334
NM_007115	TNFAIP6	2.464633
NM_001114735	BCL2A1	2.448536
NM_052941	GBP4	2.435366
NM_001012631	IL32	2.39898
NM_052864	TIFA	2.390237
NM_002463	MX2	2.389213
