# Supplementary material for: Transcriptome sequencing wide functional analysis of human mesenchymal stem cells in response to TLR4 ligand
Source: Sci Rep. 2016 Jul 22;6:30311. doi: 10.1038/srep30311 (PMC4957230; doi:10.1038/srep30311)
Supplement: Supplementary Information [file srep30311-s1.doc]

**Title: Transcriptome sequencing wide functional analysis of human mesenchymal stem cells in response to TLR4 ligand**

Sun Hwa Kim ^a^, ^†^, Amitabh Das^b, †^, Jin Choul Chai ^a, †^, Bert Binas ^a^, Mi Ran Choi ^a^, Kyoung Sun Park ^a^, Young Seek Lee ^a^, Kyoung Hwa Jung ^b^ **, Young Gyu Chai ^a, c^, *

^a^*Department of Molecular & Life Science, Hanyang University, Ansan, 15588, Republic of Korea*

^b^*Institute of Natural Science & Technology, Hanyang University, Ansan, 15588, Republic of Korea*

^c^*Department of Bionanotechnology, Hanyang University, Seoul, 04673, Republic of Korea*

^†^ These authors contributed equally to this work.


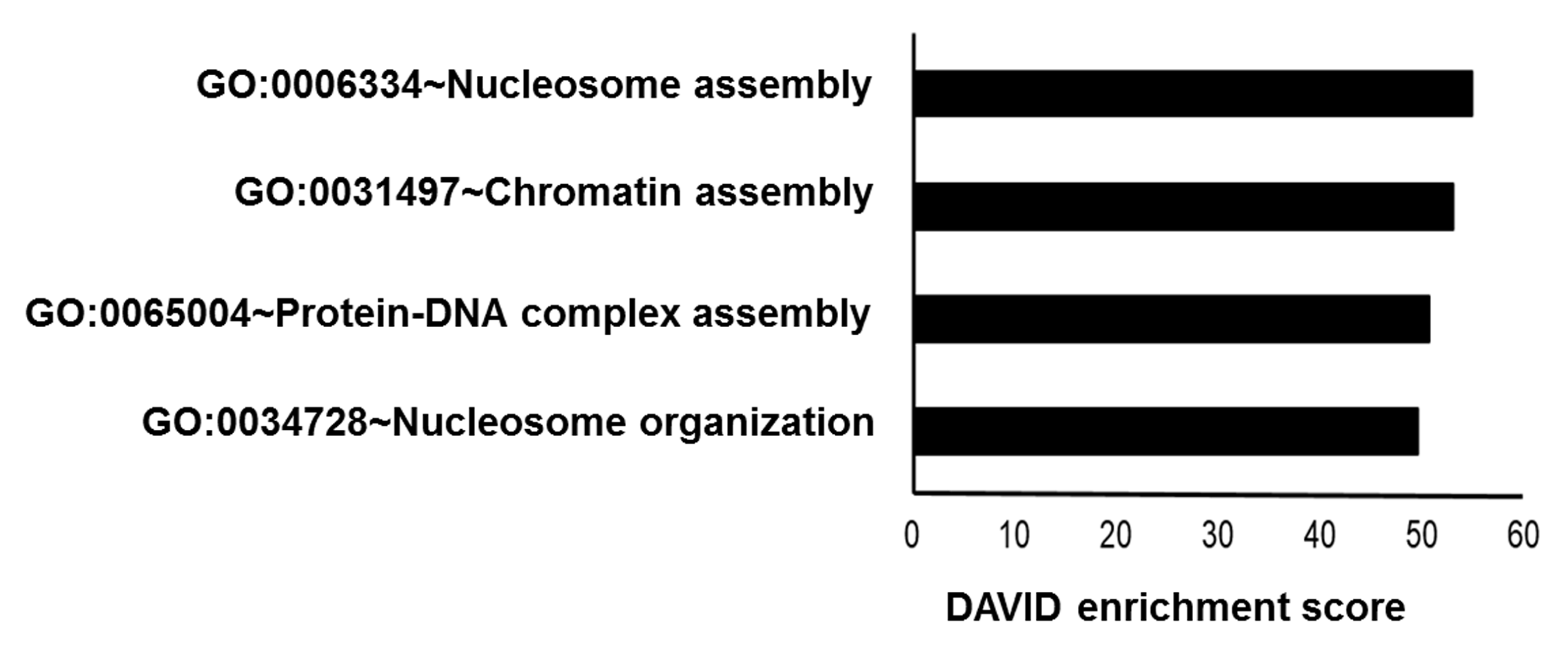


**Figure S1 Functional annotations of TLR4-primed down-regulated genes.** Gene Ontology analysis of functional annotations (biological process) associated with 4 hr TLR4-primed hMSCs down-regulated genes in comparison with the control.


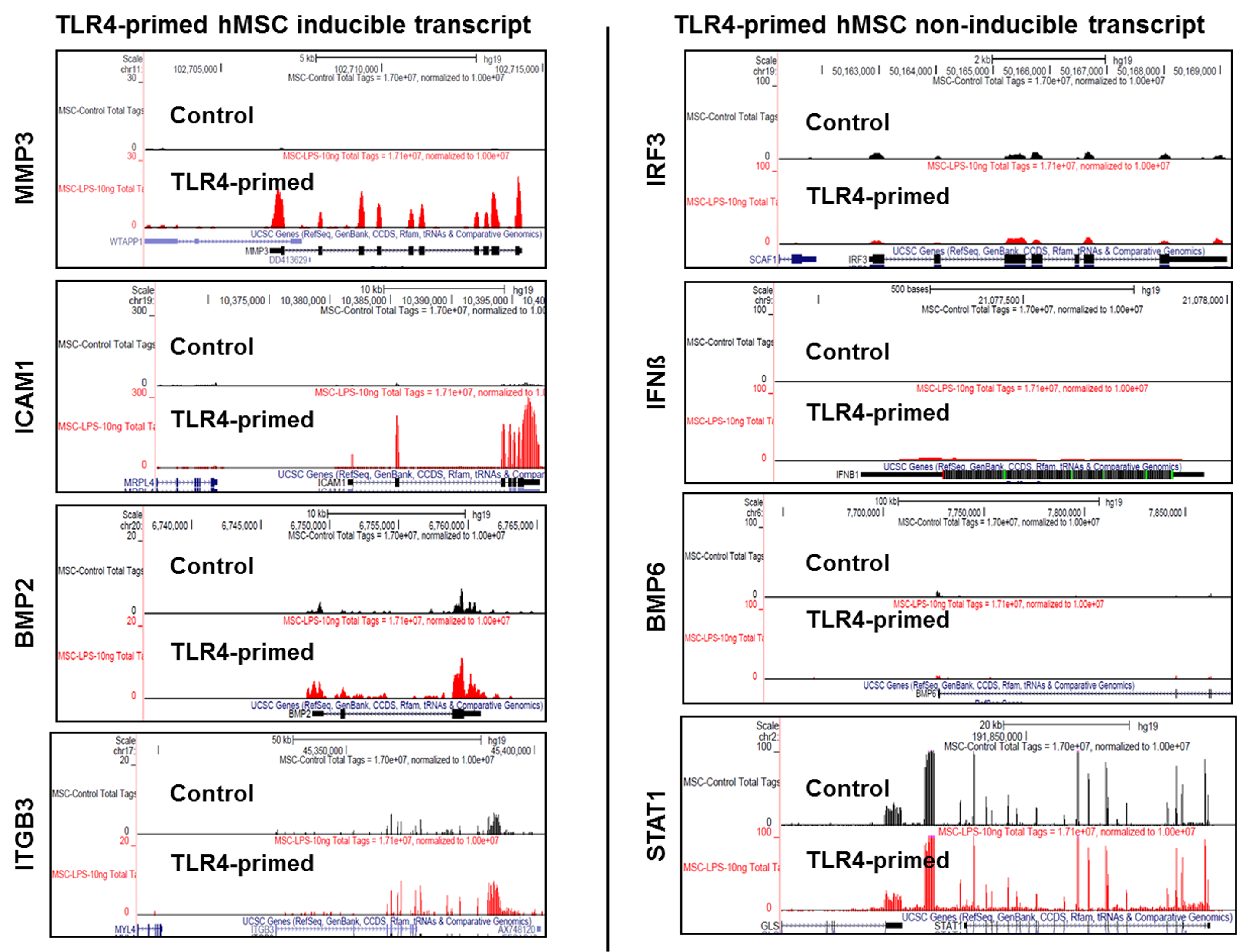


**Figure S2** **Expressions of selected transcripts in TLR4-primed hMSCs.** UCSC browser images representing normalized RNA-seq read densities of TLR4-primed inducible (left panel) and non inducible (right panel) transcript in hMSCs compared with controls.


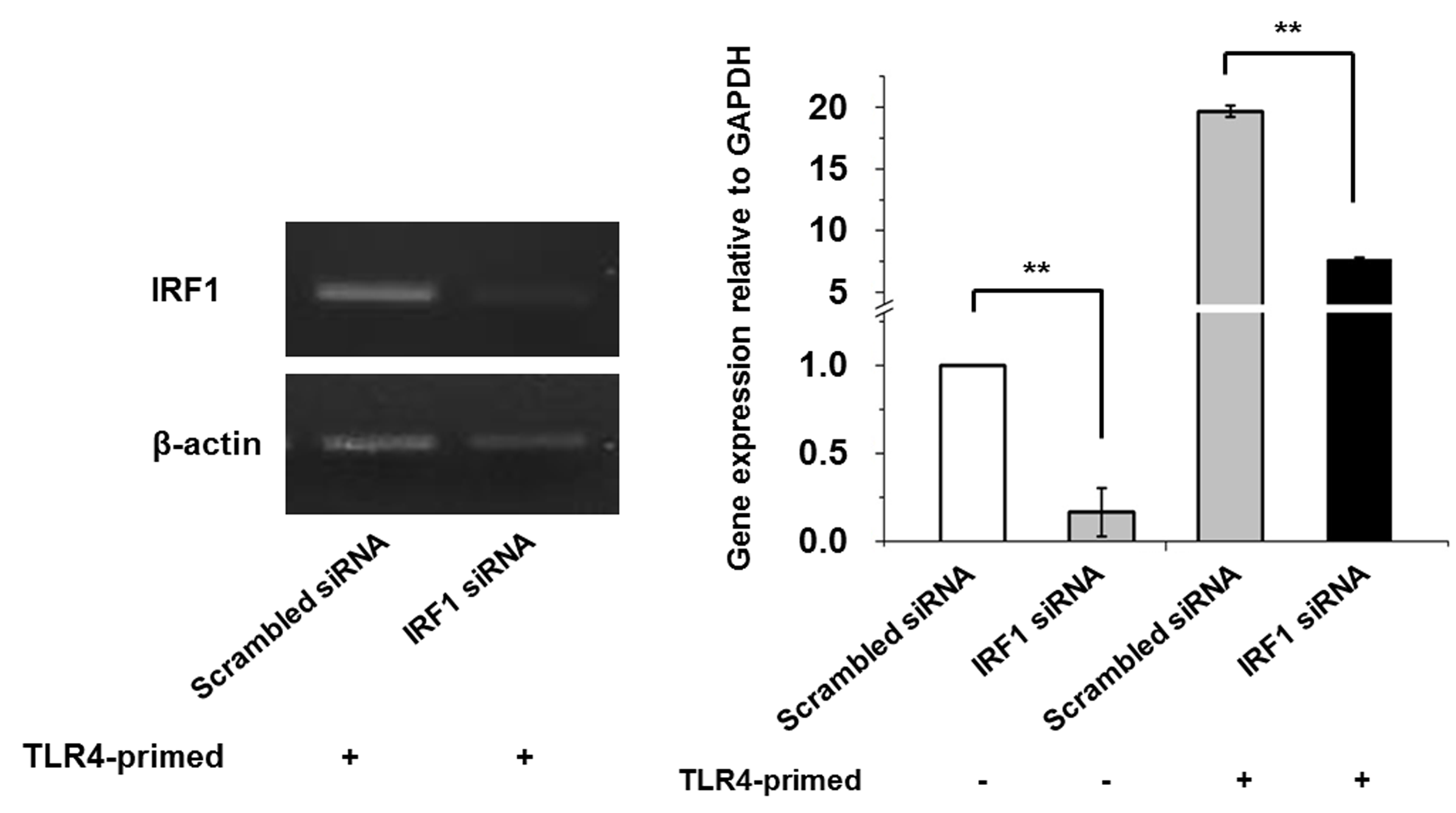


**Figure S3.** Representative RT-PCR blot and real time RT-PCR quantification showing relative mRNA expression levels of IRF1 in scrambled siRNA control (n = 3) and IRF1 siRNA treated hMSCs for 48 hr (n = 3).

**Supporting Information Table 1:** IRF1 binding motif transcript (*P* ≤ 0.001, and fold change ≥ 1.0 log_2_) in TLR4-primed hMSCs

| **IRF1 binding motif (score >=0.786696)** | | | | | |
| --- | --- | --- | --- | --- | --- |
| **Gene_ID** | **Gene symbol** | **Score** | **Position** | **Sequence (5' -> 3')** | **Strand** |
| NM_001547 | IFIT2 | 0.968059 | -4 | TTCTAGTTTCACTTTCCCTTT | + |
| NM_032789 | PARP10 | 0.949997 | -3 | TTTCAGTTTCACTTTTGTTTT | + |
| NM_030641 | APOL6 | 0.949679 | -24 | TGTCACTTTCAGTTTCCTTTT | - |
| NM_001270930 | IFIT1 | 0.946947 | -45 | CTTTAGTTTCACTTTCCCCTT | + |
| NM_001031683 | IFIT3 | 0.936883 | -7 | CATCAGTTTCACTTTCCTTTC | + |
| NM_001267706 | CD274 | 0.934967 | -169 | TTTCACTTTCTGTTTCATTTC | + |
| NM_000585 | IL15 | 0.931465 | -29 | TTTCTCTTTCACTTTTCTTTT | + |
| NM_005101 | ISG15 | 0.930121 | -82 | CTTCAGTTTCGGTTTCCCTTT | - |
| NM_024119 | DHX58 | 0.92831 | -4 | CCTCAGTTTCAGTTTCCATTT | + |
| NM_001144925 | MX1 | 0.926296 | 6 | ATTCAGTTTCGGTTTCATTTC | - |
| NM_001256765 | IL15RA | 0.919468 | -5 | ATTCGCTTTCGCTTTTGCTTT | - |
| NM_003141 | TRIM21 | 0.91888 | 6 | TTTCACTTTCAGTTTCCGCTC | - |
| NM_006187 | OAS3 | 0.91671 | -107 | CCCTGCTTTCAGTTTCGTTTC | - |
| NM_000600 | IL6 | 0.91545 | -217 | CTTTACTTTCTTTTTTTCTTT | - |
| NM_002053 | GBP1 | 0.912896 | 19 | AAGTACTTTCAGTTTCATATT | + |
| NM_001012967 | DDX60L | 0.912334 | -33 | AGTTAGTTTCGGTTTCTTTTC | - |
| NM_017631 | DDX60 | 0.909007 | -38 | CTTAGGTTTCAGTTTCCCTTC | - |
| NM_052941 | GBP4 | 0.908969 | -779 | CTTTATTTTCACTTTCAATAT | + |
| NM_006417 | IFI44 | 0.907081 | -27 | ACTGAGTTTCAGTTTCCTTTC | + |
| NM_003641 | IFITM1 | 0.901643 | -876 | AGAAACGAAAGC | + |
| NM_001002264 | EPSTI1 | 0.895371 | -473 | TTTTGATTTCAGTTTCCATTT | + |
| NM_022168 | IFIH1 | 0.895148 | -2 | CTTTGGTTTCTGTTTCGATTC | - |
| NM_001145645 | TNFSF13B | 0.893696 | 7 | TTTCACTTTCAGTTTTTGTAA | - |
| NM_000593 | TAP1 | 0.893613 | -615 | AAGCGCTTTCGCTTTCACTCT | - |
| NM_018284 | GBP3 | 0.892853 | -4 | AAGTACTTTCAGTTTCATTAG | + |
| NM_080657 | RSAD2 | 0.891621 | -39 | TTCAACTTTCAGTTTCACATG | + |
| NM_003745 | SOCS1 | 0.891269 | -134 | CTCCACTTTTGGTTTCTCTTT | - |
| NM_002038 | IFI6 | 0.885806 | -99 | CTGCAGTTTCATTTTCCCCTC | - |
| NM_001146106 | PARP9 | 0.885029 | 0 | GCAAAGTTTCAGTTTCGCTTC | + |
| NM_138287 | DTX3L | 0.885029 | -11 | GCAAAGTTTCAGTTTCGCTTC | - |
| NM_001710 | CFB | 0.884652 | -336 | TGCTTGTTTCACTTTCACATG | + |
| NM_024625 | ZC3HAV1 | 0.883703 | -5 | GATCGCTTTTAGTTTCTCTTC | + |
| NM_022147 | RTP4 | 0.883393 | -42 | GTTCAGTTTCTGTTTCTGTGT | - |
| NM_017554 | PARP14 | 0.881839 | -20 | TGACTCTTTCGCTTTCGTTTC | - |
| NM_001098478 | HLA-F | 0.88164 | -724 | TCTTTCTTTCTTTTTTTTTTT | - |
| NM_001190943 | TNFSF10 | 0.877924 | -148 | AGCTTCTTTCAGTTTCCCTCC | + |
| NM_006167 | NKX3-1 | 0.87648 | -15 | CACCGCTTTCACTTTCCGCCT | - |
| NM_001256575 | ENC1 | 0.872569 | -717 | TTCTAATTTCATTTTCCTTTA | - |
| NM_002387 | MCC | 0.870184 | -287 | TTTTTCTTTTTCTTTTCTTTT | + |
| NM_001172632 | OLR1 | 0.867795 | -221 | TTTGAATTTCAGTTTCTGTCT | + |
| NM_018370 | DRAM1 | 0.867416 | -598 | TTTCTTTTTCATTTTTTTTTT | + |
| NM_001127714 | HIVEP3 | 0.866544 | -950 | TTTGTTTTTCACTTTTCTTTT | - |
| NM_020433 | JPH2 | 0.866217 | -318 | ATTCCCTTTCTCTTTCTCCTG | - |
| NM_014314 | DDX58 | 0.861219 | -27 | CGCTAGTTGCACTTTCGATTT | + |
| NM_001199280 | HAS3 | 0.858705 | -577 | TTTTGGTTTTTTTTTTTTTTT | + |
| NM_001165 | BIRC3 | 0.857234 | -467 | ATTTATTTTTACTTTTTGTTC | - |
| NM_001199622 | NCOA7 | 0.857154 | -18 | ATTGAGTTTCTGTTTCCCTCA | - |
| NM_002800 | PSMB9 | 0.856607 | -337 | GGCCGCTTTCGATTTCGCTTT | - |
| NM_012454 | TIAM2 | 0.855703 | -142 | TCTTGATTTCTCTTTCAGATT | + |
| NM_017523 | XAF1 | 0.854901 | -758 | TTTTCATTTCATTTTTCTTTT | + |
| NM_002981 | CCL1 | 0.853932 | -929 | TTTTCCTTTTTGTTTTTTTTT | - |
| NM_001278736 | CCL5 | 0.852688 | -121 | TTTCAGTTTTCTTTTCCGTTT | + |
| NM_001565 | CXCL10 | 0.850401 | -886 | CAACACTTTTTTTTTCTTTTT | + |
| NM_022135 | POPDC2 | 0.84943 | -536 | GCTCACTTTTCCTTTCTCTCT | + |
| NM_052864 | TIFA | 0.849258 | -477 | TTTTTTTTTCTTTTTTTCTTT | - |
| NM_001077654 | TNFAIP8 | 0.846305 | -443 | CTGCTCTTTTGCTTTTAATTT | - |
| NM_003733 | OASL | 0.845777 | -329 | TTTTTGTTTTTTTTTTTTTTT | - |
| NM_001570 | IRAK2 | 0.845688 | -743 | TTTTCCTTTTTTTTTTTTTTT | + |
| NM_001142523 | IRAK3 | 0.845307 | -743 | TTCTTTTTTCAGTTTTTTTCT | + |
| NM_024873 | TNIP3 | 0.845094 | -409 | AATTATTTTCCCTTTTATTTC | + |
| NM_001200 | BMP2 | 0.845014 | -772 | TTCAACTTTCAGTTTGAAGTT | - |
| NM_001130140 | ERAP2 | 0.844889 | -395 | TTTTCCTTTTCTTTTCTTTTT | - |
| NM_001079821 | NLRP3 | 0.843317 | -315 | CCTTAGTTTCACTTCCCTCAC | + |
| NM_002090 | CXCL3 | 0.84141 | -450 | TGCCTCTCTCATTTTCGGTTT | - |
| NM_017414 | USP18 | 0.841386 | -63 | CCTCGCTTTTTTTTTTTTTTT | - |
| NM_001066 | TNFRSF1B | 0.841378 | -28 | GCGGGCTTTCGCTTTCAGTCG | + |
| NM_002309 | LIF | 0.840939 | -52 | CTTCACTCTCACTTTCTTCCA | + |
| NM_001114735 | BCL2A1 | 0.840364 | -34 | TTAAACTTTCTCTTTCATACA | + |
| NM_005226 | S1PR3 | 0.839894 | -883 | TTTTTCTTTAATTTTCTCCTT | + |
| NM_001127361 | RNF19B | 0.838156 | -719 | TTTGCCTTTCTTTTTCTAGTT | - |
| NM_199186 | BPGM | 0.83743 | -520 | CCTTACTTTCTCTTACCGCTT | - |
| NM_000874 | IFNAR2 | 0.836726 | -912 | ATTTTCTTTTTTTTTTCGTTT | + |
| NM_020226 | PRDM8 | 0.835496 | -805 | TCTTCCTCTCTCTTTCTCTCT | + |
| NM_002535 | OAS2 | 0.833668 | -107 | AGTTAGTTTTGGTTTCCCTGC | + |
| NM_003046 | SLC7A2 | 0.832024 | -335 | TTGTGGTTTCTGTTTTTGCTA | + |
| NM_002852 | PTX3 | 0.831798 | -388 | GACCATTTTCAGTTTCAACCT | + |
| NM_003629 | PIK3R3 | 0.829366 | -523 | TCTTTCTTTCTTTTTTTTCCT | + |
| NM_052862 | RCSD1 | 0.828539 | -299 | GCCTTCTTTCTTTTTCCTGTC | - |
| NM_005409 | CXCL11 | 0.828317 | -65 | CAGTGCTTTCACATTCTTATC | + |
| NM_006734 | HIVEP2 | 0.828198 | -242 | AGCTTGTTTCACTTTATATTG | + |
| NM_006820 | IFI44L | 0.826451 | -51 | TGCTGCTTTGAGTTTCAGGTT | + |
| NM_001130046 | CCL20 | 0.82599 | -897 | TGATCCTTTTACTTTCAACCT | + |
| NM_002422 | MMP3 | 0.825869 | -828 | CTTTCTTTTCTGTTTCTTTGT | - |
| NM_003108 | SOX11 | 0.825738 | -948 | CTCTGCTCTCCTTTTCCCTTT | - |
| NM_001005404 | YPEL2 | 0.824011 | -696 | ATTTTCTTTTAATTTTATTAT | + |
| NM_001271186 | RAP2C | 0.823766 | -893 | ATTTACTTCCTTTTTCCCAAT | + |
| NM_005923 | MAP3K5 | 0.823307 | -269 | GCCTCGTTTCTCTTTCGATCG | - |
| NM_001143818 | SERPINB2 | 0.823145 | -890 | TTTTTATTTTATTTTTTGTTT | + |
| NM_001024844 | CD82 | 0.821914 | -333 | CGCCTCCTTCACTTTCTCCTT | - |
| NM_145637 | APOL2 | 0.821068 | -923 | TTCTACTTTTAGTTTTTTAAG | - |
| NM_000450 | SELE | 0.820552 | -453 | TTTTTCTTCCTTTTTCACCTG | - |
| NM_001135241 | AKR1C2 | 0.819617 | -473 | TCATATTTTCTTTTTTATTTA | + |
| NM_016584 | IL23A | 0.817559 | -424 | TCTCTGTTTTGCTTTTTCCTC | + |
| NM_001104558 | ELOVL7 | 0.816871 | -549 | CTCTAGTTTCTTATTCCTTAT | + |
| NM_001424 | EMP2 | 0.814759 | -379 | CCCCTGTCTCAGTTTCCTCTT | - |
| NM_016816 | OAS1 | 0.814715 | -478 | TGCTTTTTTCCCTTTCAATTA | - |
| NM_002993 | CXCL6 | 0.813067 | -294 | TTTGACTTCCTCTTTTCCTAA | + |
| NM_001142651 | NEURL1B | 0.812666 | -743 | GATCTGTTTTATTTTCTTCCT | + |
| NM_001037335 | HELZ2 | 0.812654 | 1 | AGGAGCTCTCAGTTTCGATTC | - |
| NM_005082 | TRIM25 | 0.811463 | -515 | TTCTTCTTCTTTTTTCTTTTT | - |
| NM_004148 | NINJ1 | 0.810778 | -699 | GTTTGGTCTCTGTTTCTCCAT | - |
| NM_001270507 | TNFAIP3 | 0.810351 | -367 | GTTTTCTTTCTTTCTTATTTC | + |
| NM_007115 | TNFAIP6 | 0.809702 | -443 | GCATACTTTTTGTTTTAAATT | + |
| NM_000594 | TNF | 0.808278 | -162 | CTTCTGTCTCGGTTTCTTCTC | - |
| NM_001561 | TNFRSF9 | 0.808027 | -736 | GAATTCTTTTTTTTTTTTTTT | + |
| NM_001198 | PRDM1 | 0.807832 | -602 | TGTTTGTTTTCCTTTCCTCTC | - |
| NM_000582 | SPP1 | 0.807745 | -105 | TGTGCGTTTTTGTTTTTTTTT | + |
| NM_021105 | PLSCR1 | 0.807535 | -235 | TCTTGCTCTTTCTTTCCTTCT | - |
| NM_000295 | SERPINA1 | 0.807398 | -657 | GGCCTGTTTCTGTTTTTGCTC | - |
| NM_000228 | LAMB3 | 0.806585 | -499 | GGGATTTTTCATTTTTTTTTT | + |
| NM_001127183 | CFLAR | 0.806386 | -429 | CTTTACATCCATTTTCTTGTT | + |
| NM_005534 | IFNGR2 | 0.806135 | -931 | TTTTTTTTTTTTTTTTTTTTT | + |
| NM_001256477 | CMPK2 | 0.806101 | 4 | CTTCGCTTTCGTTTCCCGCTG | + |
| NM_001144966 | NEDD4L | 0.804415 | -447 | TTTTTGTTCCTGTTTTTTTCC | + |
| NM_001511 | CXCL1 | 0.80275 | -528 | TCTTATTTTCAGTTTAGCCTA | - |
| NM_001271003 | TFPI2 | 0.802457 | -636 | TTCTTCTTCTTCTTTTTCTTC | + |
| NM_001270531 | WTAP | 0.80205 | -458 | GAGTACTTCCACCTTCCCTTC | + |
| NM_005195 | CEBPD | 0.799775 | -380 | TCCTGGTTTTGATTTCACTCC | + |
| NM_001270691 | SMOX | 0.79924 | -613 | TCTTTGCTTCAGTTTCTTCAT | - |
| NM_182757 | RNF144B | 0.799126 | -623 | ATTTTGTTACATTTTTGTTTA | + |
| NM_033240 | PML | 0.798813 | -15 | CTTCTGTCTCACTTCCTCTCC | + |
| NM_002427 | MMP13 | 0.798657 | -125 | GGCGACTTTTTCTTTTCCCTC | - |
| NM_001190945 | TRAF1 | 0.798308 | -533 | GAACCGTTTCTGTTTTCGTCT | - |
| NM_001243835 | STAT4 | 0.796532 | -430 | TACTATTTTTACTTTCTTAAA | + |
| NM_002201 | ISG20 | 0.795992 | -730 | CTTTGGTTTTATTTTTGAGGT | + |
| NM_033035 | TSLP | 0.795941 | -122 | TACTCCTTTTTTTTTTTCCTT | + |
| NM_002426 | MMP12 | 0.795404 | -236 | GTTCTCCTTCTCTTTTAGTTG | + |
| NM_032413 | C15orf48 | 0.795316 | -855 | CTCGGGTTTTTGTTTTATTGT | + |
| NM_006291 | TNFAIP2 | 0.794553 | -309 | GGGGACTTTCACTTTACTTGG | - |
| NM_004972 | JAK2 | 0.79215 | -580 | CTCCGCTTTCGGCTTTTCCTT | - |
| NM_003811 | TNFSF9 | 0.791779 | -172 | CTCTCCTTTCCGTTTCCGGCC | - |
| NM_000584 | CXCL8 | 0.79082 | -790 | GTTCTCTTTCATCTTCCTCTA | + |
| NM_001172771 | IL34 | 0.790733 | -475 | GTTTTGTTTCGTTTTTTTAGA | + |
| NM_002994 | CXCL5 | 0.790234 | -336 | CTTTGGTTCCACCTTTTCTCT | + |
| NM_001288718 | STAT5A | 0.789057 | -641 | TACCTCTCCCAGTTTCATTCT | - |
| NM_001004419 | CLEC2D | 0.788912 | -60 | GCTTTTTTTTTGTTTTGTTTT | + |
| NM_001161572 | MAFF | 0.78848 | -948 | TCTGAATTTTTTTTTTTTTTT | - |
| NM_025106 | SPSB1 | 0.788145 | -41 | GGCGCTTTTCTCTTTTTTTTC | + |
| NM_003897 | IER3 | 0.788086 | -307 | CCTTTTTTTTTTTTTTTTTTT | - |
| NM_000710 | BDKRB1 | 0.787738 | -139 | CTCTCATTTCACTTTGCACTT | - |
| NM_002928 | RGS16 | 0.786825 | -533 | TTTAGCTTTCTGATTCTCGAT | + |
| NM_003028 | SHB | 0.786696 | -739 | GTTTGCTTACCTTTTCACCTC | - |

**Supporting Information Table 2:** TFs predicted to be activated transcript (*P* ≤ 0.001, and fold change ≥ 1.0 log_2_) in TLR4-primed hMSCs

| **NFkB1 predicted to be activated  (p=5.19 e-34)** | | **IRF1 predicted to be activated (p=5.62 e-33)** | |
| --- | --- | --- | --- |
| **ID** | **Fold Change** | **ID** | **Fold Change** |
|  | **(≥ 1.0 log_2_-fold)** |  | **(≥ 1.0 log_2_-fold)** |
| TSLP | 2.951 | VCAM1 | 3.336 |
| TNFAIP3 | 2.350 | TRIM21 | 1.386 |
| SDC4 | 1.093 | TNFSF10 | 1.034 |
| PTGS2 | 1.157 | TNF | 1.043 |
| POU2F2 | 1.291 | TAP1 | 1.794 |
| MMP3 | 3.438 | SOCS1 | 1.068 |
| MMP13 | 1.151 | RSAD2 | 1.367 |
| MAP3K8 | 2.666 | PTGS2 | 1.157 |
| IL6 | 2.672 | PSMB9 | 1.124 |
| IL23A | 1.771 | PML | 1.264 |
| IER3 | 2.053 | OAS1 | 2.694 |
| GBP1 | 2.003 | MX1 | 2.785 |
| CXCL8 | 5.629 | JAK2 | 1.020 |
| CXCL11 | 1.250 | ISG15 | 3.199 |
| CXCL10 | 3.056 | IL6 | 2.672 |
| CSF2 | 2.253 | IL15 | 1.062 |
| CSF1 | 2.938 | IFITM1 | 1.789 |
| CCL5 | 3.798 | IFIT3 | 4.067 |
| BMP2 | 1.103 | IFIT2 | 4.409 |
| BIRC3 | 2.172 | IFIT1 | 3.627 |
| BCL2A1 | 2.450 | IFIH1 | 1.678 |
| TNF | 1.043 | IFI44L | 1.010 |
| SOD2 | 3.576 | GBP2 | 1.595 |
| CXCL3 | 4.611 | CXCL11 | 1.250 |
| CD82 | 1.908 | CXCL10 | 3.056 |
| CCL2 | 2.810 | CD274 | 1.723 |
| BAMBI | 1.334 | CCL5 | 3.798 |
| CCL20 | 3.566 | CCL2 | 2.810 |
| CFB | 1.831 | CFB | 1.831 |
| CX3CL1 | 1.370 | CXCL8 | 5.629 |
| CXCL2 | 3.823 | OAS2 | 2.625 |
| IFNGR2 | 1.051 |  |  |
| ISG15 | 3.199 |  |  |
| PTX3 | 1.391 |  |  |
| SELE | 3.323 |  |  |
| TNFSF10 | 1.034 |  |  |
| TRAF1 | 3.785 |  |  |
| VCAM1 | 3.336 |  |  |

**Supporting Information Table 3: List of primers used in qRT-PCR studies.**

| **Gene symbol** | **Forward Sequence (5' -> 3')** | **Reverse Sequence (5' -> 3')** |
| --- | --- | --- |
| **CCL1** | CTCATTTGCGGAGCAAGAGAT | GCCTCTGAACCCATCCAACTG |
| **CCL2** | CAGCCAGATGCAATCAATGCC | TGGAATCCTGAACCCACTTCT |
| **CCL3** | AGTTCTCTGCATCACTTGCTG | CGGCTTCGCTTGGTTAGGAA |
| **CCL5** | CAGCACGTGGACCTCGCACA | GGCAGTGGGCGGGCAATGTA |
| **CCL7** | TGCTCAGCCAGTTGGGATTA | GCTACTGGTGGTCCTTCTGT |
| **CXCL1** | AGGCAGGGGAATGTATGTGC | AGCCCCTTTGTTCTAAGCCA |
| **CXCL8** | CAAACCTTTCCACCCCAAAT | CTCAGCCCTCTTCAAAAACT |
| **CXCL10** | GGAAGGTTAATGTTCATCATCCTAAGC | TAGTACCCTTGGAAGATGGGAAAG |
| **FGF2** | AGTGTGTGCTAACCGTTACCT | ACTGCCCAGTTCGTTTCAGTG |
| **GAPDH** | AAGGTCGGAGTCAACGGATT | CTCCTGGAAGATGGTGATGG |
| **IFIH1** | TCGAATGGGTATTCCACAGACG | GTGGCGACTGTCCTCTGAA |
| **IFIT1** | TTGATGACGATGAAATGCCTGA | CAGGTCACCAGACTCCTCAC |
| **IFIT2** | AAGCACCTCAAAGGGCAAAAC | TCGGCCCATGTGATAGTAGAC |
| **IFIT5** | GGCCAAAATAAAGACGCCCTT | GACCAGGCTTCGTACTTCTTC |
| **IFITM1** | CCAAGGTCCACCGTGATTAAC | ACCAGTTCAAGAAGAGGGTGTT |
| **IL1B** | CGACACCCTCGTTATCCCATGTGT | CTCCGACCACCACTACAGCAAG |
| **IL6** | CAGGAGAAGATTCCAAAGAT | CTCTTGTTACATGTCTCCTT |
| **IRF1** | ATGCCCATCACTCGGATGC | CCCTGCTTTGTATCGGCCTG |
| **IRF3** | AGAGGCTCGTGATGGTCAAG | AGGTCCACAGTATTCTCCAGG |
| **IRF7** | CCCACGCTATACCATCTACCT | GATGTCGTCATAGAGGCTGTTG |
| **ISG15** | CGCAGATCACCCAGAAGATCG | TTCGTCGCATTTGTCCACCA |
| **ITGB3** | GTGACCTGAAGGAGAATCTGC | CCGGAGTGCAATCCTCTGG |
| **NFKB1** | GAAGCACGAATGACAGAGGC | GCTTGGCGGATTAGCTCTTTT |
| **NFKB2** | ATGGAGAGTTGCTACAACCCA | CTGTTCCACGATCACCAGGTA |
| **PTGS2** | CATTCTTTGCCCAGCACTTCAC | GACCAGGCACCAGACCAAAGAC |
| **RELB** | CCATTGAGCGGAAGATTCAACT | CTGCTGGTCCCGATATGAGG |
| **TNF-α** | ATGAGCACTGAAAGCATGATCC | GAGGGCTGATTAGAGAGAGGTC |

**Supporting Information Table 4: List of primers used in ChIP-PCR studies.**

| **Name** | **Forward Sequence (5' -> 3')** | **Reverse Sequence (5' -> 3')** |
| --- | --- | --- |
| IFITM1 Region_1 | CAGGGACTCCAAGCAAGGG | AACCATCAGCCCCTCCCTAC |
| IFITM1 Region_2 | TTGTAATTCATGATCCTTGTGCCT | TTCACAGGGACGGGGAATTGT |
| IFITM1 Region_3 | AGGTCTCTCCTGCATGGCTA | GACTCCCCAGAGGGTAGTGT |
| IFITM1 Region_4 | GCCCCCTAAACCCAACACTT | CTTTATGGCAGGGTGGGAGG |
| IFIT2 Region_1 | ATCACAAGGCAGGGAGCTTT | TGCACAAGGGCTGGTAAACT |
| IFIT2 Region_2 | AGTTGTGCTCCCTTGATGCC | GAGTAAGAGCCAAGCCCAGC |
| IFIT2 Region_3 | CACAACTCAGCTCCGGAGGAA | GTGGCCTCTGGTTCCTTTTTGTTT |
| IFIT2 Region_4 | CCGAACAGCTGAGAATTGCAC | CTGGCCCTCTTTGGGAACATA |
| IFIH1 Region_1 | CCTTTGGTTAAGGACACCGC | GAGCTTTGCTAGAGCGTTGG |
| IFIH1 Region_2 | GGAGGCCTTTGGTTAAGGAC | CTGTTCACTTCCCCTTTGGA |
| IFIH1 Region_3 | CCAAAGGGGAAGTGAACAGA | CCCACAGGAGGTCAGGTTTA |
| IFIH1 Region_4 | CCTCGGCTGGTGAATGTAGG | AGTGTTTGGGAACCCACAGG |
